# Performance Evaluation of Regional Water Environment Integrated Governance: Case Study from Henan Province, China

**DOI:** 10.3390/ijerph17072501

**Published:** 2020-04-06

**Authors:** Ran He, Zhen Tang, Zengchuan Dong, Shiyun Wang

**Affiliations:** 1 School of Business, Hohai University, Nanjing 210000, China; tangzh@hhu.edu.cn; 2 College of Hydrology and Water Resources, Hohai University, Nanjing 210000, China; zcdong@hhu.edu.cn (Z.D.); w18851811580@163.com (S.W.)

**Keywords:** water environment integrated governance, improved entropy-weight TOPSIS model, obstacle degree, performance evaluation, Henan Province

## Abstract

The performance of the regional water environment integrated governance is affected by many factors. This study took place in Henan Province, China, as the research area, and constructed an index system through the comprehensive consideration of three target layers based on the Ecological-Social-Economic (ESE) framework. Due to advantages such as strong objectivity and operability, the improved entropy-weight technique for the order of preference by similarity to the ideal solution (TOPSIS) model can greatly overcome subjective human interference and render the evaluation results more reliable. Therefore, it was introduced to evaluate the water environment integrated governance in Henan from 2007 to 2016. By applying the obstacle degree model, the obstacle factors were then diagnosed. The results of this study show that the overall performance of the integrated governance was generally improved in Henan from 2007 to 2016. Performance levels of the three target layers exhibited different trends, of which the social and economic benefits presented a linear increase year by year, but the ecological benefits presented a fluctuating downward trend. The obstacle on the Henan water environment integrated governance mainly comes from the ecological and economic benefits index. Therefore, a series of countermeasures have been proposed as a means of improving the governance performance in Henan.

## 1. Introduction

With rapid economic and social development, conflicts between human beings and nature have been increasing. The unreasonable development and utilization of water resources, as well as the discharging of sewage, have brought severe challenges to the sustainable development of society [1]. Water resources, as a carrier of human survival, are of great significance to humanity but are being increasingly threatened by factors such as the urbanization, agricultural intensification, and climate change [2]. Since the 1960s, China’s environment has suffered various degrees of damage, among which the destruction of water resources is the most serious [3]. According to authoritative data, in 2016, 60% of the country’s total surface water quality was polluted at moderate to high levels, 298 million rural residents drank water that was unsafe, and 20% of urban residents drank substandard water [4]. As an important strategic measure to alleviate the water pollution and resolve complex water problems, water environment governance is particularly important. In 2017, the 19th National Congress of the Communist Party of China pledged to “adhere to the harmonious coexistence of humanity and nature” as the basic strategy for upholding and developing socialism with Chinese characteristics in the new era. To promote the construction of ecological civilization, the governance of environmental pollution must be accelerated as soon as possible. In this study, research into the issue of water environment integrated governance has gradually become a focal point. The formulation of a scientific and reasonable mechanism to evaluate the performance of water environment integrated governance is currently a significant issue in both the theory and practice of water environment improvement.

The shortage and serious pollution of water resources are severe obstacles to meeting the demand for clean water [5,6]. Academic research on the water environment governance from different perspectives has been emerging in an endless stream [7]. At present, the research mainly includes the vulnerability of water resources [8], evaluation of water quality [9], and methods of urban water governance [10]. The construction of evaluation indexes has gradually changed from the traditional viewpoint of ‘water ecological’ to the more comprehensive evaluation of ecology, society and economics [11,12]. For example, Fekete and Stakhiv studied the establishment of such indicators for a performance evaluation index system, and outlined a conceptual framework for indicators suitable to guiding both the water management planning as well as implementation evaluation [13]. On the basis of the ILBM (Lake Basin Management Initiative), Chidammodzi and Muhandiki built an indicator system from two aspects, namely the water resource subsystems and socioeconomic subsystems for the evaluation of water environment governance in the Lake Malawi Basin [14].

The existing research provides a certain reference for the evaluation of water environment integrated governance, but there are also several problems and shortcomings we need to take into account. The index systems have been constructed mainly based on the river basin water environment governance, but there is still a lack of performance evaluation index systems and methods for the regional water environment integrated governance. The performance evaluation is dependent on the comprehensive consideration of the effectiveness and impact of water environmental governance on changes in water quality and policy implementation. In fact, the performance of water environment governance shows a dynamic trend corresponding to the degree of economic and social development. In order to solve these problems, we propose a practical method to evaluate the performance of water environment integrated governance. The main contributions of this paper are as follows—first, a water environment integrated governance evaluation index system was constructed, through the comprehensive consideration of three target layers comprising ecological, social, and economic benefits. Second, an entropy weight method was used to determine the index comprehensive weight. Finally, the performance of water environment integrated governance in Henan Province, China, from 2007 to 2016, was evaluated by using the improved technique for the order of preference by similarity to the ideal solution (TOPSIS) model, and obstacles to the performance were diagnosed by applying the obstacle degree model. The method can objectively evaluate the integrated governance level of water environment in Henan Province or even the whole of China.

## 2. Materials and Methods

### 2.1. Study Area Background

**Figure 1:**
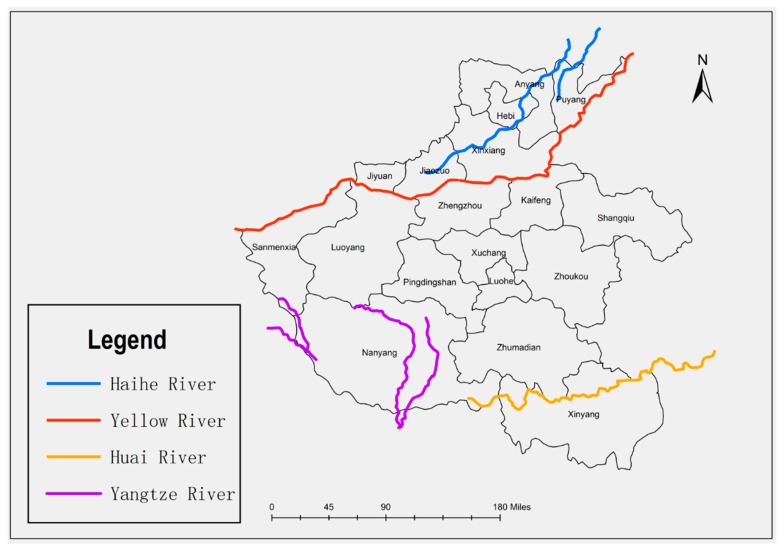
Four major river basins in Henan Province, China.

Henan Province is located in the eastern part of China, belonging to the humid and semihumid regions. Spanning the Hai River, Yellow River, Huai River, and Yangtze River (Figure 1), it is the source of Huai River and Middle Route Project which is part of the South-to-North Water Transfer Project of China. The total amount of water resources in Henan Province is 41.3 billion cubic meters, ranking the 19th among all the provinces of China. As one of the most important economic regions of China, a large amount of industrial and domestic wastewater has caused serious water pollution. According to data from the year 2001, the total discharge of wastewater in Henan Province was about 2.4 billion tons, of which 47.0% was industrial wastewater and 53.0% was domestic sewage. According to the drainage area statistics, the Hai, Yellow, Huai, and Yangtze River Basins discharged 0.707, 0.401, 1.025, and 0.203 billion tons of wastewater. According to the Environmental Quality Standards for Surface Water (GB3838-2002) [15], the total length of rivers with the water quality worse than Class V was 2336 km, accounting for 50.8% of the total length of the assessed rivers. Since the introduction of China’s “sustainable development concept” in 2003, Henan Province has continued to implement water environment remediation projects. The regional water environment governance has achieved its initial success.

### 2.2. Methods

This study comprehensively considered the index systems of water environment governance from perspectives of the three dimensions of economy, society, and ecology to build a regional water environment integrated governance performance evaluation index system. The entropy-weight method was introduced to determine the weight of each indicator, improved TOPSIS, and obstacle degree models as the theoretical basis to evaluate the performance of regional water environment integrated governance in Henan Province. The method procedure is described in the following section.

#### 2.2.1. Assessment Index System

Performance, which usually refers to the efficiency and effectiveness [16], is widely used in the management, economics, and organizational behavior [17]. It is a comprehensive concept covering multiple objectives, and is often influenced by innovative technologies and resources [18]. In the governance of water environment, performance can be considered as an effect of governance, i.e., an objective and comprehensive reflection of the methods and results of governance.

**Figure 2:**
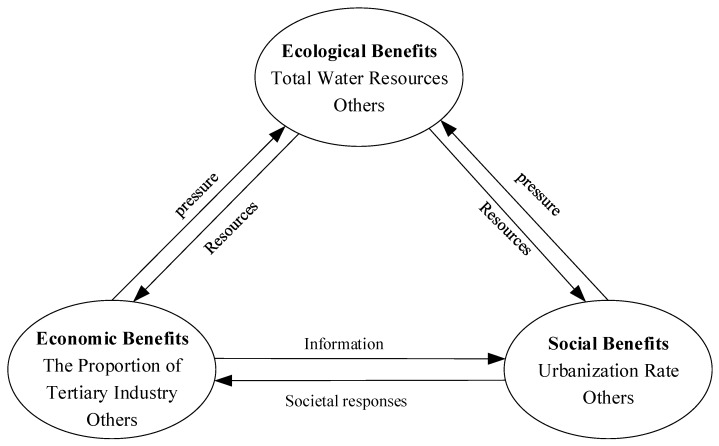
Ecological-Social-Economic (ESE) Framework.

Researchers commonly believe that it is necessary to ensure both the quality and quantity of water resources, which must be done to meet the needs of human survival and social development, particularly at a certain stage of social and economic development. It means that the regional water environment integrated governance is not only an ecological and environmental issue, but also an issue involving social and economic factors [19,20]. To this end, this study began with the three dimensions of ecological, social, and economic benefits, and then comprehensively evaluated the performance of regional water environment governance. The Ecological-Social-Economic (ESE) water environment integrated governance evaluation framework is shown in Figure 2.

In the ESE Framework, ecological benefits are mainly reflected in the enhancement of urban ecosystem functions and reduction of pollutants, which can be improved by the ecological restoration, river remediation, water quality control, and other means [21]. And it can provide resources for social benefits and economic benefits. So, wastewater treatment rate, source water quality compliance rate, river water quality compliance rate, total surface water resources, total water resources, and per capita water resources are its representative indicators. The social benefits refer to the transformation of the values of resource benefits [22], which are mainly reflected in the improvement of water quality for the residents, quality of domestic water, and carrying capacity of water resources. Investment in water environment governance projects, urbanization rate, public life satisfaction, water environment governance public satisfaction and Engel’s coefficient can belong to this dimension. Besides, the urbanization rate can be calculated by the proportion of urban population to total population, and it can both represent the proportion of people who have access to clean water and the carrying capacity of water resources to some degree. So, it is also the representative indicator of social benefit. The economic benefits are mainly reflected in the improvement of commercial values brought about by the water environment governance and increase in the industrial benefits associated with the water environment governance [21]. And unit GDP water consumption, industrial added value water consumption, per capita disposable income, gross regional product and proportion of tertiary industry can belong to this dimension. Besides, social and economic benefits can put some pressure on ecological benefits. 

Therefore, an index system of the regional water environment integrated governance performance evaluation that includes ecological, social, and economic benefits, as well as qualitative and quantitative indicators was built. And it combines the results of references [7,21,22], the expert opinion, and Water Environmental Quality Assessment Index [23] proposed in the Environmental Technology Draft Report published by the United States Environmental Protection Agency (EPA) in 2003. The specific indicator system is shown in Table 1.

#### 2.2.2. Entropy-Weight TOPSIS Method

Two of the most commonly used methods for determining index weights are the Analytic Hierarchy Process (AHP) [24,25] and Delphi methods [26,27], which are highly subjective. So, this study used the entropy-weight method to determine the indicator weights. Entropy is used to indicate the degree of the information disorder and can express the information implied by the index data, as well as avoid problems encountered in an analysis due to the inconsistency among indicators [28]. As a method of objectively determining weights, the entropy-weight method has strong objectivity and operability, thereby overcoming the subjective human interference and rendering the evaluation results more reliable. The specific calculation steps of the entropy-weight method are as follows.

(a) The initial indicator normalization process:

(1) $$f_{ij}=\frac{x_{ij}}{\sum_{j=1}^{n}x_{ij}}$$

where $x_{ij}$ is the initial value of the indicator in *j* years, and $f_{ij}$ is the normalized value of $x_{ij}$, *i = 1, 2, …, m, j = 1, 2, …, n*.

(b) The indicator entropy value calculation:

(2) $$u_{i}=-\frac{1}{\ln n}\sum_{j=1}^{n}f_{ij}\ln f_{ij}$$

where $u_{i}$ is the entropy value of the index *i*.

(c) The entropy weight can be calculated with the formula:

(3) $$w_{i}=\frac{1-u_{i}}{m-\sum_{i=1}^{m}u_{i}}$$

where $w_{i}$ is the weight of the index *i*. 

#### 2.2.3. Improved TOPSIS Model and the Performance Level Criteria

The performance levels of the integrated water environment governance change dynamically with the economic development and related technological advances. The best performance is to approach the best state of water environment governance. The TOPSIS model is an “approximate ideal solution ranking” method, which is mainly used in system engineering to solve the problem of multiobjective decision-making with finite schemes [29,30]. Compared with other methods, the TOPSIS method has no strict requirements for the indicator data, sample content, and data distribution, but can make full use of the original data in the evaluation index with less loss of information. At the same time, the method can achieve horizontal and vertical comparisons of different evaluation objects with the advantages of simple, real, and reliable operability. The weight determined by using the entropy-weight method in the present study was able to reflect the relative importance of the index more accurately [31]. In addition, this study improved the ideal solution of TOPSIS and introduced the virtual worst solution to better distinguish the merits of indicators [32,33]. The specific calculation steps are as follows.

(a) Raw data preprocessing.

Perform the same trend processing for each indicator value. The formula is as follow:

(4) $$\begin{array}{cc} \mathrm{Positive}\;\mathrm{indicators}: & x_{ij}^{\prime }=x_{ij} \end{array}$$

(5) $$\begin{array}{cc} \mathrm{Negative}\;\mathrm{indicators}: & x_{ij}^{\prime }=\frac{1}{x_{ij}} \end{array}$$

(b) Standardize the original data.

(6) $$r_{ij}=\frac{x_{ij}^{\prime }}{\sqrt{\sum_{i=1}^{m}x_{ij}^{2}}}$$

(c) Construct a weighted normalized matrix Y.

(7) $$Y=R•W={|y_{ij}|}_{mn}$$

(d) Determine the positive and negative ideal solutions, and then introduce the virtual worst solution.

(8) $$y^{+}=\left\{\max y_{ij}|i=1,2,\cdots ,m\right\}=\left\{y_{1}^{+},y_{2}^{+},\cdots ,y_{m}^{+}\right\}$$

(9) $$y^{-}=\left\{\min y_{ij}|i=1,2,\cdots ,m\right\}=\left\{y_{1}^{-},y_{2}^{-},\cdots ,y_{m}^{-}\right\}$$

(10) $$y^{*}=2y^{-}-y^{+}$$

where $y^{+}$ is the positive ideal solution, $y^{-}$ is the negative ideal solution, and $y^{*}$ is the virtual worst solution.

(e) Calculate the distance from the evaluation object to the positive ideal and virtual worst solutions. 

Distance to the positive ideal solution:

(11) $$d_{j}^{+}=\sqrt{\sum_{i=1}^{m}{\left(y_{ij}-y_{i}^{+}\right)}^{2}}$$

Distance to the virtual worst solution:

(12) $$d_{j}^{*}=\sqrt{\sum_{i=1}^{m}{\left(y_{ij}-y_{i}^{*}\right)}^{2}}$$

(f) Calculate the degree of closeness, which ranges from 0 to 1 and indicates the closeness between the evaluation indicator and the best goal. The higher the degree of closeness, the higher the performance level.

(13) $$T_{j}^{k}=\frac{d_{j}^{*}}{d_{j}^{*}+d_{j}^{+}}$$

where *j* is the year row, *j = 1, 2, …, 10*, when *k = 1, 2, 3*, indicating the relative closeness between the three target layers and ideal solution, respectively. *k = 4* signifies the total closeness among the 16 indicators and ideal solution. Divide the closeness into four levels [34]. The grading is shown in Table 2.

#### 2.2.4. Obstacle Degree Model

By analyzing and diagnosing the main obstacle factors that affect the performance of regional water environment integrated governance, countermeasures and guidelines for the governance can be formulated and adjusted accordingly. Three basic variables in the diagnostic model are introduced, including the factor contribution degree $F_{i}$, indicator skewness $I_{i}$, and obstacle degree $O_{i}$. $F_{i}$ represents the contribution of a single indicator to the overall target (water environmental governance performance) and can generally be expressed by the weight $w_{i}$ of each indicator. $I_{i}$ is the difference between the actual value of each indicator and the optimal target value. This difference can be represented by the value of $1-r_{ij}$. The obstacle degree $O_{i}$ can indicate the level of the influence of target and indicator layers on the performance of water environment governance. The formula is:

(14) $$O_{i}=\frac{I_{i}•w_{i}}{\sum_{i=1}^{m}I_{i}•w_{i}}$$

where $I_{i}=1-r_{ij}$.

### 2.3. Data Sources

The quantitative indicator data used in this paper are all from the Statistical Yearbook of Henan Province (2007–2016) published by the Henan Statistics Bureau [35], and Henan Water Resources Bulletin (2007–2016) published by the Water Resources Department of Henan Province [36]. The qualitative indicator comes from the survey data.

## 3. Results and Discussions

### 3.1. Results of Weights

According to the Equations (1)–(3), the weight of each index is calculated by using the entropy weight method, and the results are shown in Table 3.

### 3.2. Performance Evaluation of the Water Environment Integrated Governance in Henan Province

By employing the method mentioned above, as well as the data collected in Henan Province from 2007 to 2016, the performance was evaluated and the final results were provided in Table 4.

#### 3.2.1. Overall Performance Analysis

**Figure 3:**
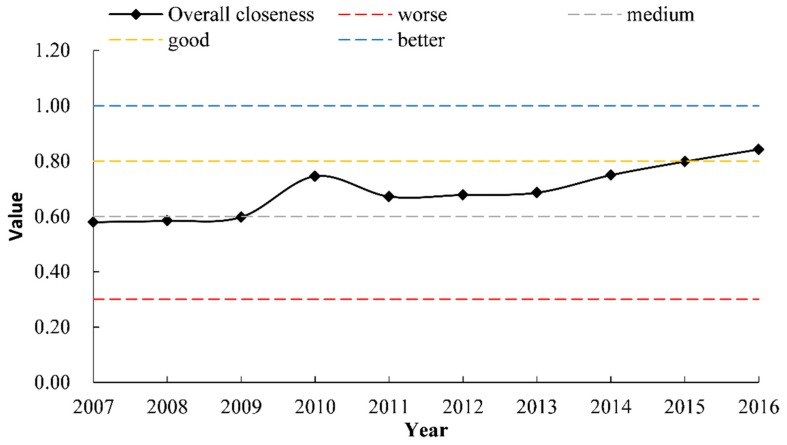
Trends in the overall performance.

The trend curve of the overall performance from 2007 to 2016 is shown in Figure 3. From Figure 3, the level of the water environment integrated governance in Henan Province generally shows an upward trend. And this can be verified by Cui with the vector norm method and radar map method [37]. In 2016, the regional water environment governance performance index reached 0.84, nearly 50% more than that in 2007. During 2007–2016, judging from the development level of the performance of Henan Province, it has experienced a process from medium to good and to excellent. Looking at the whole, the performance level went through three stages, which are described in detail below.

The first stage (2007–2009)—the performance level of the water environment integrated governance in Henan Province was at a medium level and growing at a low rate. It was at 0.60 in 2009, which was not a significant increase compared to 0.58 in 2007 (Table 4). The analysis found that the main reason was the average annual rainfall of about 700 mm across the whole province during 2007–2009, which were dry years. During this period, water resources were not abundant. However, since Henan Province is a large agricultural province, rural agriculture developed rapidly and irrigation water consumption increased significantly. The average water consumption reached 177 m^3^ per acre, resulting in an overall increase in water consumption by agriculture. Meanwhile, due to the low efficiency of agricultural use of water resources, there was a serious waste of agricultural water. 

The second stage (2010–2014)—the performance level was good and fluctuated from 0.67 to 0.75, among which the performance level was 0.74 in 2010 because the rainfall of that year was extremely high. After that, the performance level rose from 0.67 to 0.75, and the main reason for this improvement was the announcements of the “Regulations for the Prevention and Control of Water Pollution in Henan Province” in 2011. Under the guidance of these policies, the Henan provincial government spent about 35 billion RMB in planning and building the infrastructure, such as the urban sewage treatment, reclaimed water utilization, and sludge disposal infrastructure, for water environmental treatment. During this period, Henan Province built 185 municipal sewage treatment plants and 924 sewage treatment projects in rural areas, as well as directed a total of 211 administrative villages, to conduct comprehensive rural environmental improvements.

The third stage (2015–2016)—the performance level was excellent and growing from 0.81 in 2015 to 0.83 in 2016. The reason for this improvement was the promulgation of the ‘Henan Province Blue Water Project Action Plan (water pollution prevention and control work program)’ in 2015 to promote further improvements, which contributed to an increasing institutionalization and rationalization of the water environment governance, and promoted the rapid growth of the governance performance.

#### 3.2.2. Target Layer Performance Analysis

**Figure 4:**
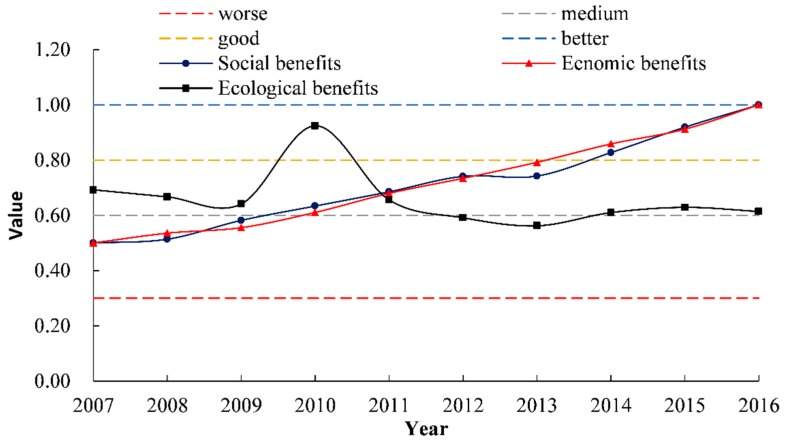
Trends in the performance of target layers.

Trends of the performance in the target layers of water environment integrated governance evaluation are shown in Figure 4, performance levels of the social and economic benefits of the governance in Henan Province from 2007 to 2016 were increasing year by year. Although the rising trends of social and economic benefits were basically similar, there were also certain differences. In 2008–2012, the growth rate of the social benefits was faster and the performance level was slightly greater than those of the economic. In the following three years (2012–2014), the growth rate and performance level of the economic benefits exceeded those of the social. In 2014–2015, performance levels of both the benefits were excellent. Performance of the ecological benefits fluctuated and showed an overall downward trend. It was declining each year in 2007–2009, but in 2010, it increased to 0.92, which was the highest of the decade. However, the performance level fell again in 2011–2013 and was only medium by 2013. In 2014–2016, it remained in a good level.

### 3.3. Diagnosis of the Obstacle Degree 

The obstacle degree of each indicator was calculated and ranked from the largest to the smallest value. For ease of analysis, only those indicators ranked as the top five obstacles were analyzed. From 2007 to 2016, the top five obstacle indicators were the total surface water resources (C4), per capita water resources (C6), investments in the water environment (C7), per capita disposable income (C14), and gross regional product (C15). The obstacle coefficients of the five obstacle indicators remained basically unchanged in the ten years mentioned above, signifying that these five indicators had a greater impact on performance of the water environment integrated governance. In contrast, the indicators that did not appear in the top five had less impact on the governance.

**Figure 5:**
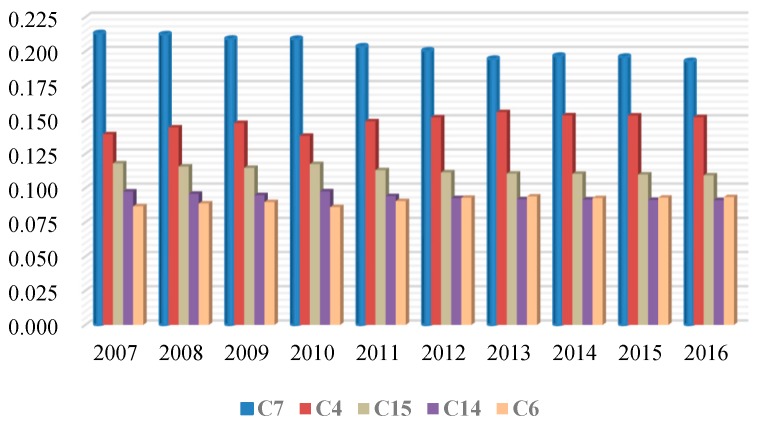
Difference of the obstacle factors’ degree from 2007 to 2016.

The difference of obstacle factors’ degree from 2007 to 2016 is shown in Figure 5. The top three indicators (investment in the water environment (C7), total surface water resources (C4), and gross regional product (C15)) in this decade were the same, which were distributed separately into social, ecological, and economic benefits. The analysis found out that from 2007 to 2016, the main obstacle that hindered the governance performance was the insufficient investment in water environment projects. Lack of investment in the water environment integrated governance was the main contributor to the failure of the performance improvement. In addition, lack of total surface water resources and gross regional products were also the main reasons constraining the performance.

**Figure 6:**
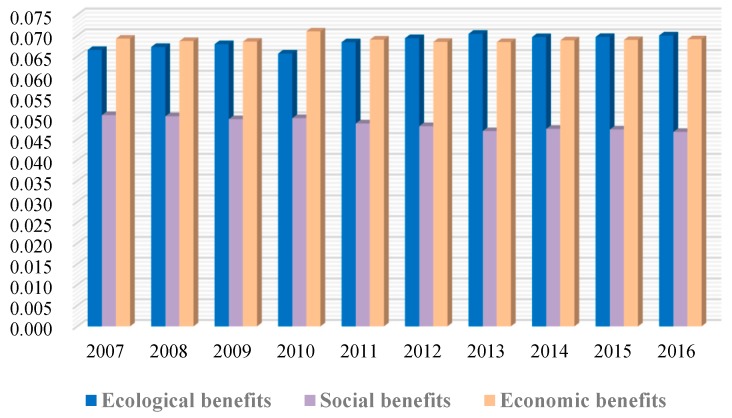
Difference of the target layers’ obstacle degrees from 2007 to 2016.

For the ecological, social, and economic benefits, the obstacle degrees were not the same for the performance from 2007 to 2016. Moreover, degrees of the ecological and economic benefits were greater than that of the social. As shown in Figure 6, in the three target layers, the social benefits had the lowest obstacle degree in the performance. From 2007 to 2011, the degree of the economic benefits was the highest. During 2012–2016, the degree of the ecological benefits exceeded that of the economic and had the greatest impact on the performance level. The obstacle degree of the ecological benefits increased slowly, yet those of the social decreased slowly, and those of the economic remained basically unchanged after small fluctuations.

## 4. Conclusions

The water environment integrated governance is an important way of improving the water environment. In this paper, a water environment integrated governance evaluation index system through the comprehensive consideration of three target layers based on the Ecological-Social-Economic (ESE) framework was first constructed. Then, due to the advantages like strong objectivity and operability, the entropy-weight method was introduced to determine the index comprehensive weight. Finally, the performance of water environment integrated governance in Henan Province, China, from 2007 to 2016, was evaluated by using the improved technique for the order of preference by similarity to the ideal solution (TOPSIS) model, and obstacle factors of the performance were diagnosed by applying the obstacle degree model. Several conclusions can be drawn as below.

(1) The improved entropy-weight TOPSIS model was used to evaluate the performance level of the water environment integrated governance in Henan Province, China, from 2007 to 2016. The level exhibited an upward trend on the whole. In 2016, the value reached 0.83. The performance level showed a process of change that was “medium-good-excellent”, meaning that the level had undergone the medium- and low-speed growth (2007–2009) to good and fluctuating growth (2010–2014), and finally, excellent and rapid growth (2015–2016).
(2) The performance evaluation results of the target layers show that the performance levels of the economic and social benefits had risen year by year, which changed from a medium (2007–2009) to a good (2010–2014) to an excellent level (2015–2016), basically consistent with the overall performance. Although general trends in the growth of both the above two benefits are basically the same, there are also some differences. And the performance level of the ecological benefits stayed at a good level but reached an excellent level in 2010.
(3) The obstacle degree analysis of the indicator layers shows that the five obstacle factors that had the greatest impact on the level of governance from 2007 to 2016 were the total surface water resources (*C4*), per capita water resources (*C6*), investment in the water environment (*C7*), per capita disposable income (*C14*), and gross regional product (*C15*). The obstacle degree analysis of the target layers shows that from 2007 to 2011, the obstacle degree of economic benefits was the highest. From 2012 to 2016, the obstacle degree of ecological benefits was the highest, and the social benefits had the lowest obstacles to the governance performance.
(4) On the basis of the results and actual situation in Henan Province, the following suggestions are put forward:
  (a) Increase the investment in water environment governance projects. The investment ought to be directly related to the effects of governance and provide a certain guarantee for the surface water resources protection, urban wastewater treatment, river water body restoration, and source water quality compliance. Therefore, our government should focus more on the investment and provide more support to guarantee the policy formulation, engineering technology, and human resources.
  (b) Use scientific methods to protect surface water resources. On the one hand, increase the total quantity of water resources, build water projects such as dams and reservoirs according to the local conditions, store excess surface water during the high flow season, and prevent the loss of surface water resources. On the other hand, protect the quality of water resources by protecting the environment along river banks and lakes, strengthen the planting of vegetation to prevent the flow of water and soil, prohibit the untreated discharge of industrial wastewater and domestic sewage, and prevent the water environment pollution.
  (c) Promote the coordinated development of economy and water environment. On the one hand, the policies for water environment control should be proposed implemented, in order to strictly supervise the discharge of sewage from industry and agriculture, and improve the efficiency of water environment control. On the other hand, abandon the rough economic development route and improve technology develop a conservation-oriented, environmentally friendly, and green economy.

In general, the evaluation model for water environment integrated governance proposed in this paper not only obtains the evaluation level of comprehensive management of water environment in Henan, but also clarifies the focus of work for future water environment improvement and construction through obstacle analysis. The evaluation model coordinates three targets of an ecological, social, and economic nature. It can be widely applied to reflect the scientific and systematic character of water environment integrated governance, which also has important reference significance for areas in addition to Henan. 

## Figures and Tables

**Table 1:** Evaluation index system of the regional water environment integrated governance.

Target Layer	Indicators Layer	Unit	Index Character
Ecological benefits	Wastewater treatment rate C_1_	%	+
	Source water quality compliance rate C_2_	%	+
	River water quality compliance rate C_3_	%	+
	Total surface water resources C_4_	10^9^ m^3^	+
	Total water resources C_5_	10^9^ m^3^	+
	Per capita water resources C_6_	m^3^/person	+
Social benefits	Investment in water environment governance projects C_7_	10^9^ Yuan	+
	Urbanization rate C_8_	%	+
	Public life satisfaction C_9_	/	+
	Water environment governance public satisfaction C_10_	/	+
	Engel’s coefficient C_11_	/	−
Economic benefits	Unit GDP water consumption C_12_	m^3^/10^4^ Yuan	−
	Industrial added value water consumption C_13_	m^3^	-
	Per capita disposable income C_14_	10^5^ Yuan	+
	Gross regional product C_15_	10^9^ Yuan	+
	Proportion of tertiary industry C_16_	%	+

**Table 2:** Performance level criteria.

Closeness	Performance Level
[0, 0.3]	Worse
(0.3, 0.6]	Medium
(0.6, 0.8]	Good
(0.8, 1.0]	Excellent

**Table 3:** Weights of evaluation indicators for the water environment integrated governance.

Target layer	Indicator	Entropy Value	Entropy Weight
Ecological benefits	*C*1	0.9877	0.0713
	*C*2	0.9998	0.0009
	*C*3	0.9975	0.0142
	*C*4	0.9744	0.1484
	*C*5	0.9852	0.0854
	*C*6	0.9843	0.0908
Social benefits	*C*7	0.9650	0.2028
	*C*8	0.9975	0.0144
	*C*9	0.9982	0.0106
	*C*10	0.9992	0.0046
	*C*11	0.9980	0.0113
Economic benefits	*C*12	0.9869	0.0759
	*C*13	0.9899	0.0586
	*C*14	0.9838	0.0939
	*C*15	0.9805	0.1131
	*C*16	0.9994	0.0036

**Table 4:** Results of the water environment integrated governance performance evaluation in Henan Province (2007–2016).

Criteria Layer	2007	2008	2009	2010	2011	2012	2013	2014	2015	2016
Ecological benefits	0.69	0.67	0.64	0.92	0.65	0.59	0.56	0.61	0.63	0.61
Social benefits	0.50	0.51	0.58	0.63	0.69	0.74	0.74	0.83	0.92	1.00
Economic benefits	0.50	0.54	0.56	0.61	0.68	0.73	0.79	0.86	0.91	1.00
Overall closeness	0.58	0.58	0.60	0.74	0.67	0.68	0.69	0.75	0.81	0.84
